# Supplementary figures and images for: De novo prediction of RNA 3D structures with deep generative models
Source: PLoS One. 2024 Feb 15;19(2):e0297105. doi: 10.1371/journal.pone.0297105 (PMC10868834; doi:10.1371/journal.pone.0297105)

**a****Nucleotide Encoding****G: 1, C:2, A:3, U:4**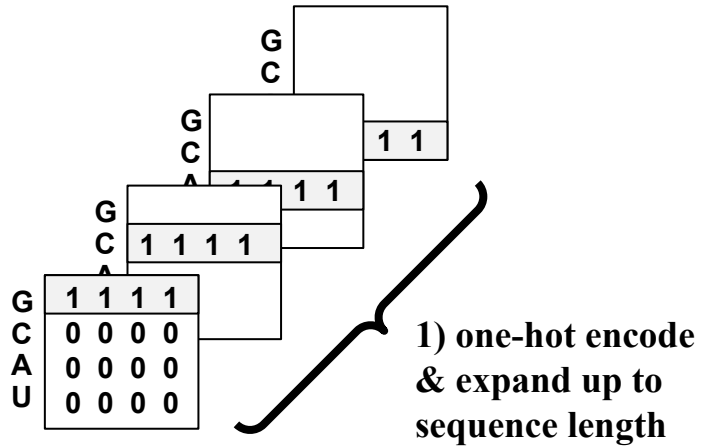**b**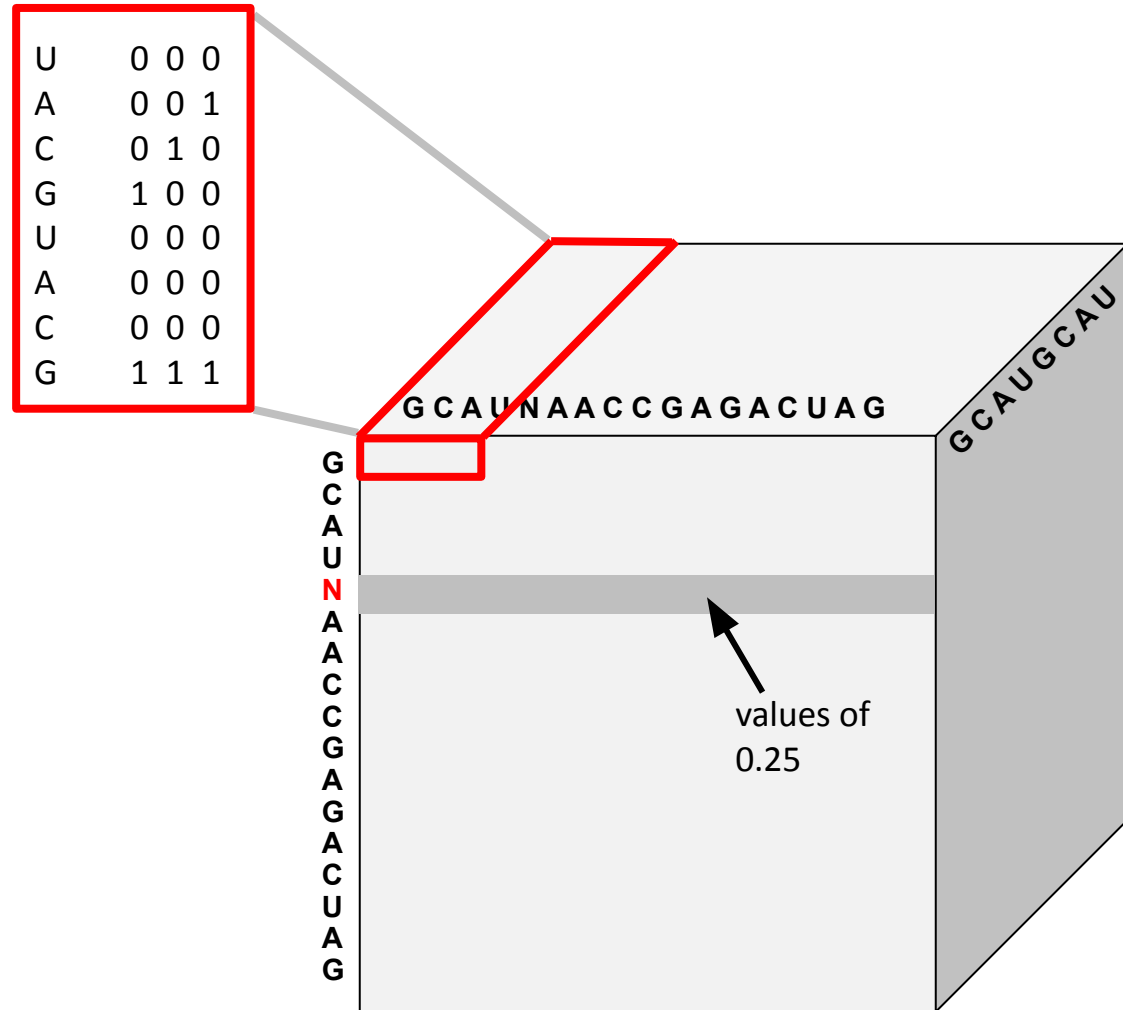

Supplement: S1 Fig — RNA sequences of length L were encoded as unique bit patterns of shape (L × L × 8): A First, every nucleotide in an RNA sequence was one-hot encoded, e.g. G: (1, 0, 0, 0), C: (0, 1, 0, 0), A: (0, 0, 1, 0), U: (0, 0, 0, 1), N: (0.25, 0.25, 0.25, 0.25). For the full sequence, these one-hot encodings led to a tensor of shape (L × 4). Unknown nucleotides that were denoted by an “N” in the sequence were encoded by setting all values in the one-hot encoding to 0.25. This one-hot encoded sequence was then copied L times (a1) to obtain a tensor of shape (L × L × 4). Then, this tensor and its transpose (a2) were stacked along the last dimension to obtain a tensor of shape (L × L × 8). b A sample sequence Tensor that corresponded to a unique bit pattern for each possible pairing and also contained directional information. For sequences with L < 100, the sequence tensor was uniformly padded with −1 Red insert: example bit pattern for the Tensor at the first three pixels with depth 8. (PDF) [file pone.0297105.s001.pdf]

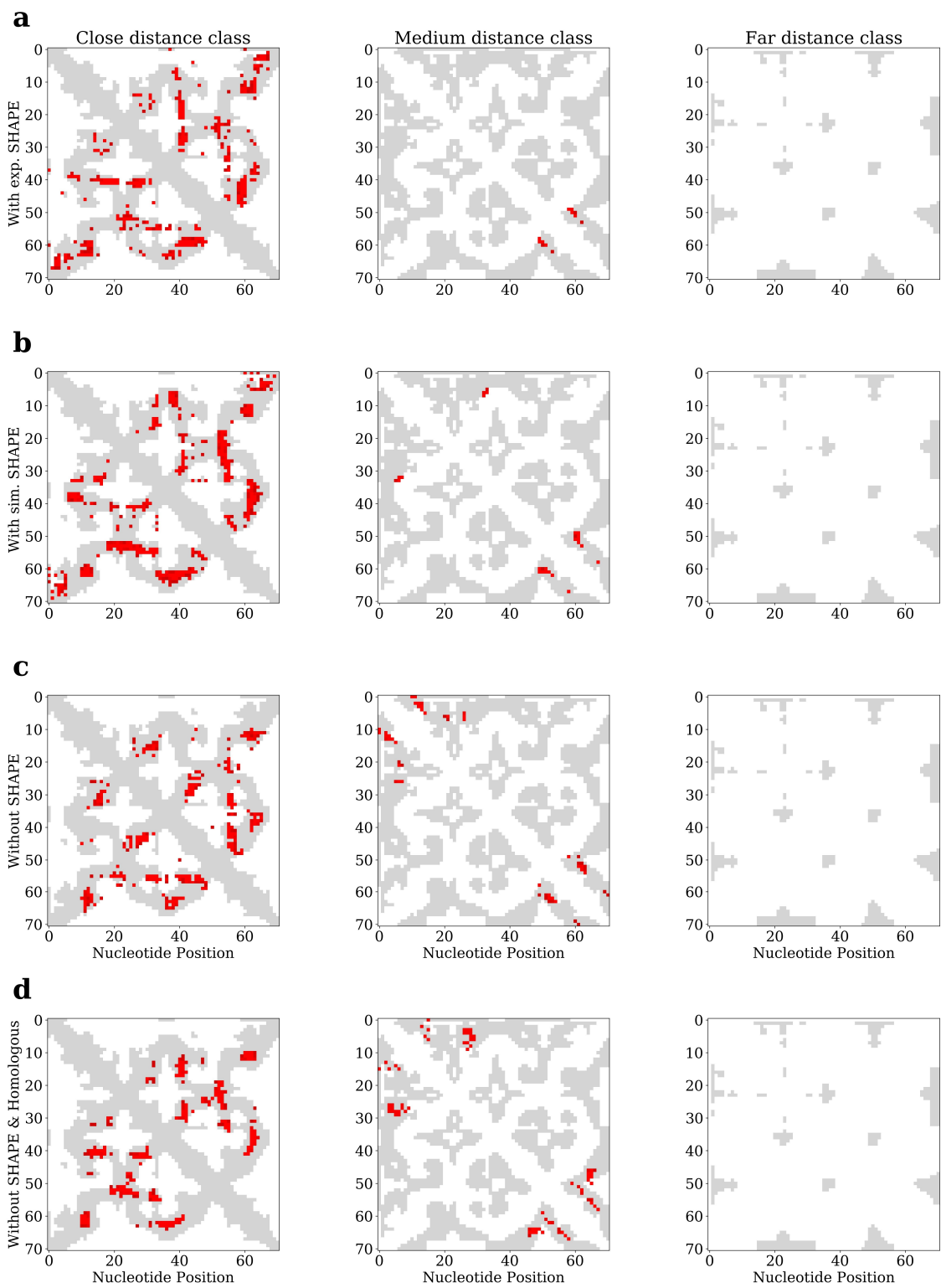

Supplement: S2 Fig — Attention Maps were a good indicator for the location of structural contacts. High attention values (red pixels) were almost exclusively found at the “near” distance class, resulting in higher probability scores for the generator’s initial prediction for that class. Incorporation of experimental A or simulated B SHAPE data both resulted in lower false positive rates of attention placement compared to when no SHAPE data C or no SHAPE data and no homolgous sequence information d was used, with false positive rates being, 4.3%, 5.7%, 14.8% and 21.4%, respectively (the false positive rate was calculated as the number of incorrectly placed red, high attention points divided by the total number of red, high attention points, where high attention points were defined by attention scores above 0.01). (PDF) [file pone.0297105.s002.pdf]

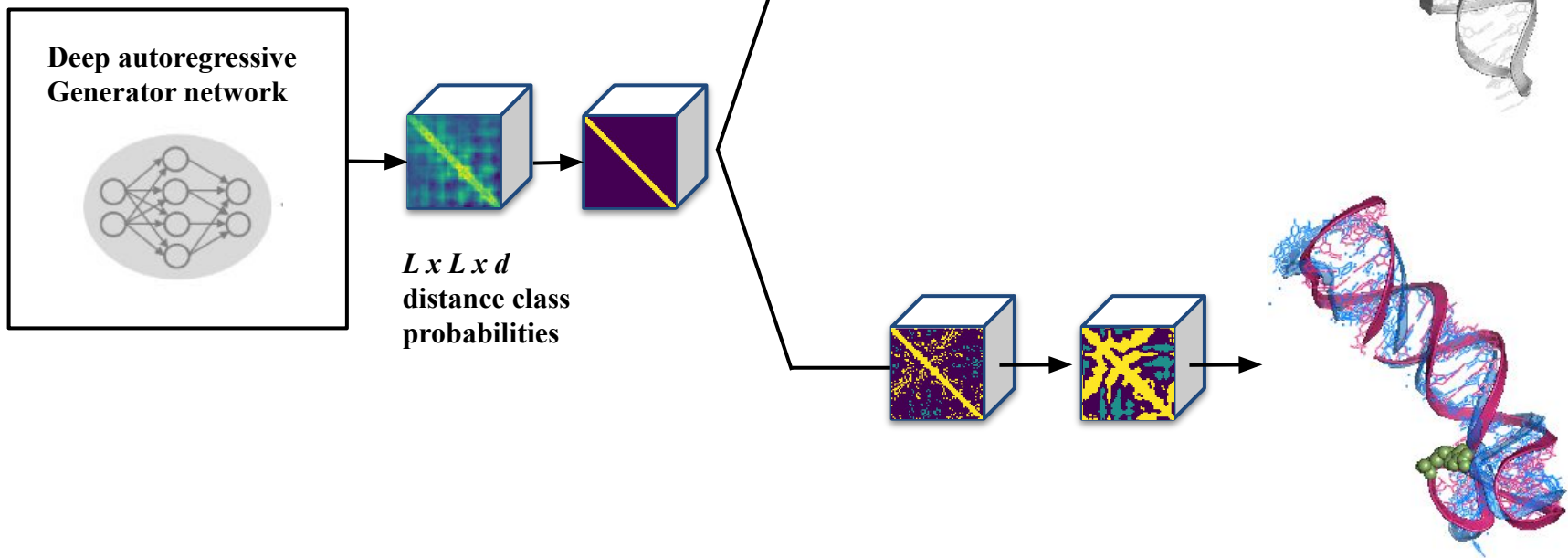

Supplement: S3 Fig — Starting with all target distance classes masked, the Generator places initial probabilities for every pixel in the distance class softmax prediction. From there, pixels were sampled iteartively using the MCTS search objective which aimed for entropy reduction. We derived two terminal leaf nodes A for which the Generator network saw enough distance pixels to fill up the remainers using its argmax prediciton B. From those filled up leafs, the VQ-VAE could be applied to decode into real distance space C. After further energy refinement, we showed for the ZMP-Riboswitch (PDB: 4XW7), that those two leafs indeed corresponded to two different structures. The riboswitch has a movable hinge part, which gets stabilized by a small molecule. Hence, our best leaf prediction in red is closer to the blue target solution, bottom C. We also sampled an alternative structure. In this particular example, the strechted grey RNA structure C was ranked with a lower score by the Score Model. (PDF) [file pone.0297105.s003.pdf]
